# The status of MRI databases across the world focused on psychiatric and neurological disorders

**DOI:** 10.1111/pcn.13717

**Published:** 2024-08-20

**Authors:** Saori C. Tanaka, Kiyoto Kasai, Yasumasa Okamoto, Shinsuke Koike, Takuya Hayashi, Ayumu Yamashita, Okito Yamashita, Tom Johnstone, Franco Pestilli, Kenji Doya, Go Okada, Hotaka Shinzato, Eri Itai, Yuji Takahara, Akihiro Takamiya, Motoaki Nakamura, Takashi Itahashi, Ryuta Aoki, Yukiaki Koizumi, Masaaki Shimizu, Jun Miyata, Shuraku Son, Morio Aki, Naohiro Okada, Susumu Morita, Nobukatsu Sawamoto, Mitsunari Abe, Yuki Oi, Kazuaki Sajima, Koji Kamagata, Masakazu Hirose, Yohei Aoshima, Sayo Hamatani, Nobuhiro Nohara, Misako Funaba, Tomomi Noda, Kana Inoue, Jinichi Hirano, Masaru Mimura, Hidehiko Takahashi, Nobutaka Hattori, Atsushi Sekiguchi, Mitsuo Kawato, Takashi Hanakawa

**Affiliations:** ^1^ Brain Information Communication Research Laboratory Group Advanced Telecommunications Research Institutes International Kyoto Japan; ^2^ Division of Information Science Nara Institute of Science and Technology Nara Japan; ^3^ Department of Neuropsychiatry, Graduate School of Medicine The University of Tokyo Tokyo Japan; ^4^ The International Research Center for Neurointelligence (WPI‐IRCN) The University of Tokyo Institutes for Advanced Study (UTIAS) Tokyo Japan; ^5^ University of Tokyo Institute for Diversity & Adaptation of Human Mind (UTIDAHM) Tokyo Japan; ^6^ Center for Brain Imaging in Health and Diseases (CBHD) The University of Tokyo Hospital Tokyo Japan; ^7^ Department of Psychiatry and Neurosciences, Graduate School of Biomedical and Health Science Hiroshima University Hiroshima Japan; ^8^ Center for Evolutionary Cognitive Sciences, Graduate School of Art and Sciences The University of Tokyo Tokyo Japan; ^9^ Laboratory for Brain Connectomics Imaging RIKEN Center for Biosystems Dynamics Research Hyogo Japan; ^10^ Department of Brain Connectomics Kyoto University Graduate School of Medicine Kyoto Japan; ^11^ Graduate School of Information Science and Technology The University of Tokyo Tokyo Japan; ^12^ Center for Advanced Intelligence Project RIKEN Tokyo Japan; ^13^ School of Health Sciences Swinburne University of Technology Melbourne Victoria Australia; ^14^ Department of Psychology, Department of Neuroscience, Center for Perceptual Systems, Center for Learning and Memory The University of Texas at Austin Austin Texas USA; ^15^ Neural Computation Unit Okinawa Institute of Science and Technology Graduate University Okinawa Japan; ^16^ Department of Neuropsychiatry, Graduate School of Medicine University of the Ryukyus Okinawa Japan; ^17^ Biomarker R&D department SHIONOGI & CO., Ltd Osaka Japan; ^18^ Department of Neuropsychiatry Keio University School of Medicine Tokyo Japan; ^19^ Hills Joint Research Laboratory for Future Preventive Medicine and Wellness Keio University School of Medicine Tokyo Japan; ^20^ Neuropsychiatry, Department of Neurosciences Leuven Brain Institute, KU Leuven Leuven Belgium; ^21^ Geriatric Psychiatry University Psychiatric Center KU Leuven Leuven Belgium; ^22^ Medical Institute of Developmental Disabilities Research Showa University Tokyo Japan; ^23^ Graduate School of Humanities Tokyo Metropolitan University Tokyo Japan; ^24^ Department of Psychiatry and Behavioral Sciences, Graduate School of Medical and Dental Sciences Tokyo Medical and Dental University Tokyo Japan; ^25^ Department of Psychiatry Haryugaoka Hospital Fukushima Japan; ^26^ Department of Psychiatry Aichi Medical University Aichi Japan; ^27^ Department of Psychiatry, Graduate School of Medicine Kyoto University Kyoto Japan; ^28^ Department of Human Health Sciences Kyoto University Graduate School of Medicine Kyoto Japan; ^29^ Integrative Brain Imaging Center National Center of Neurology and Psychiatry Tokyo Japan; ^30^ Department of Neurology, Graduate School of Medicine Kyoto University Kyoto Japan; ^31^ Department of Radiology Juntendo University School of Medicine Tokyo Japan; ^32^ Department of Integrated Neuroanatomy and Neuroimaging Kyoto University Graduate School of Medicine Kyoto Japan; ^33^ Research Center for Child Mental Development Chiba University Chiba Japan; ^34^ Research Center for Child Mental Development University of Fukui Fukui Japan; ^35^ Department of Stress Sciences and Psychosomatic Medicine, Graduate School of Medicine The University of Tokyo Tokyo Japan; ^36^ Department of Behavioral Medicine, National Institute of Mental Health National Center of Neurology and Psychiatry Tokyo Japan; ^37^ Student Counseling Center Meiji Gakuin University Tokyo Japan; ^38^ Center for Brain Integration Research Tokyo Medical and Dental University Tokyo Japan; ^39^ Department of Neurology Juntendo University Graduate School of Medicine Tokyo Japan; ^40^ Neurodegenerative Disorders Collaborative Laboratory RIKEN Center for Brain Science Saitama Japan

**Keywords:** data sharing, database, MRI, neurological disorders, psychiatric disorders

## Abstract

Neuroimaging databases for neuro‐psychiatric disorders enable researchers to implement data‐driven research approaches by providing access to rich data that can be used to study disease, build and validate machine learning models, and even redefine disease spectra. The importance of sharing large, multi‐center, multi‐disorder databases has gradually been recognized in order to truly translate brain imaging knowledge into real‐world clinical practice. Here, we review MRI databases that share data globally to serve multiple psychiatric or neurological disorders. We found 42 datasets consisting of 23,293 samples from patients with psychiatry and neurological disorders and healthy controls; 1245 samples from mood disorders (major depressive disorder and bipolar disorder), 2015 samples from developmental disorders (autism spectrum disorder, attention‐deficit hyperactivity disorder), 675 samples from schizophrenia, 1194 samples from Parkinson's disease, 5865 samples from dementia (including Alzheimer's disease), We recognize that large, multi‐center databases should include governance processes that allow data to be shared across national boundaries. Addressing technical and regulatory issues of existing databases can lead to better design and implementation and improve data access for the research community. The current trend toward the development of shareable MRI databases will contribute to a better understanding of the pathophysiology, diagnosis and assessment, and development of early interventions for neuropsychiatric disorders.

## Introduction

### Call for large‐scale databases across specialties

Precision medicine, which is an individually tailored medical approach based on the individual's biological information, has revolutionized decision, treatment, and interventions across many areas of medicine. This is a departure from the “one‐fit‐all” strategy toward the “tailor‐made” strategy for medical practice. When we turn our eyes toward psychiatry, research and practice have been made primarily with the Diagnostic and Statistical Manual of Mental Disorders (DSM)^1^ system based on signs and symptoms only. Although the DSM system allows clinicians to put a patient into a category in most cases, problems exist in terms of heterogeneity, comorbidity, and over‐specification of the diagnosis. These make it difficult to gain proper understanding of the pathophysiology of mental disorders, thereby decreasing motivations for developing novel drugs for mental disorders.^2^ The recognition of such problems stemming from the current diagnostic system has motivated the Research Domain Criteria (RDoC) initiative. The RDoC aims to redefine and identify subtypes of psychiatric disorders from the viewpoint of neurobiology, not solely on symptoms and signs.^3^ This initiative is expected to extend our understanding of the heterogeneous and overlapping clinical presentations of psychiatric disorders and overlapping pathophysiology.^4^, ^5^, ^6^, ^7^ Moreover, in the aged population, the border between psychiatric and neurological disorders is ambiguous as exemplified by dementia with Lewy body (DLB) which often presents at onset with psychiatric symptoms only. To advance the RDoC concept, it is thus essential to construct a large‐scale database including not only multiple psychiatric disorders but also some neurological disorders. This task is not as easy as it may initially sound because clinician‐scientists, who are supposed to be the primary driver of such activity, have their own specialty: schizophrenia, mood disorders, eating disorders, developmental disorders, or dementia.

Recent successes of machine learning techniques have been remarkable in the neuroimaging research for psychiatric disorders.^8^, ^9^, ^10^, ^11^, ^12^, ^13^ For example, existing studies presented a classification of psychiatric disorders based on functional MRI (fMRI),^14^, ^15^, ^16^, ^17^ subtyping of neurological and psychiatric disorders,^8^, ^14^, ^18^, ^19^, ^20^, ^21^, ^22^ and investigation of biological relationships among multiple psychiatric disorders.^17^, ^19^, ^23^, ^24^ For producing replicable findings, however, it is important that machine learning classifiers learn from large MRI datasets, ideally 1000 to 10,000, if not more.^25^ The enrollment of such large numbers of participants is unrealistic for a single clinical site. Therefore, construction of a large‐scale brain imaging database of psychiatric disorders will likely require large‐scale collaborations among multiple imaging centers and that will require data sharing, and database infrastructure.

A large‐scale, multi‐center, multi‐disorder database is important to make neuroimaging‐based knowledge useful to clinical practice everywhere. This is because an increasing number of studies have highlighted the difficulty in generalization of neuroimaging markers of neuropsychiatric disorders built from a single database to data acquired from different imaging centers.^26^, ^27^, ^28^ Accordingly, it has become important to verify reliability and reproducibility of neuroimaging markers, so that the markers can be applicable to any MRI system at any imaging center in any country.^29^

Recently, there has been movement toward a more open model of scientific practice than before – a practice called “open science.” Open science promotes open access publishing, open data, or data sharing, and sharing of methods including code. It also promotes communicating scientific findings with the public. The power of open science has just been demonstrated. As an emergency measure for the COVID‐19 pandemic, research funders and journal publishers reached an agreement that scientific articles would not be accepted unless data are shared with the World Health Organization at the time of submission (https://wellcome.org/press-release/sharing-research-data-and-findings-relevant-novel-coronavirus-ncov-outbreak, https://wellcome.org/press-release/publishers-make-coronavirus-covid-19-content-freely-available-and-reusable). As a consequence, a large number of open datasets related to COVID‐19 were registered on data archives (ex. https://data.cdc.gov/browse?tags=covid-19). It is widely believed that scientific and medical progress during the COVID‐19 pandemic was dramatically advanced because of this agreement.^30^

Open science is the key to develop neuroimaging markers generalizable to any MRI system at any imaging center in any country. Indeed, the movement toward neuroscience data sharing has dramatically progressed over the past few years^31^ driven in part by the International Brain Initiative (IBI, https://www.internationalbraininitiative.org). IBI is an international collaborative effort of national projects launched in 2018, including Canada, South Korea, Europe, the United States, Australia, China, and Japan. The IBI has established several working groups including the Data Standards and Sharing Working Group, which facilitates discovery, harmonization, and use of data across the borders. The discussion sought to create short‐ and long‐term action plans and resulted in a position paper on international data governance (https://www.internationalbraininitiative.org/news/international-data-governance-neuroscience), providing organized considerations in data sharing at each stage of the data life cycle, such as collection, processing, curation, archiving and preservation, application, and use, sharing, and deletion. These considerations must be taken into account to develop large‐scale, multi‐center, multi‐disorder MRI databases.

### Data harmonization is necessary for large‐scale, multi‐center, multi‐disorder studies

Since the effect of imaging centers is widely known as a confounding factor in neuroimaging data,^32^, ^33^ development of a method to harmonize brain imaging data between different centers is fundamental and urgent. Harmonization methods can be broadly divided into two: prospective harmonization and retrospective harmonization. In retrospective harmonization, differences in data across centers are removed using statistical methods, including deep learning.^34^, ^35^, ^36^, ^37^, ^38^, ^39^, ^40^, ^41^, ^42^, ^43^ In prospective harmonization, a standard imaging protocol is pre‐determined to allow for data acquisition with a wide range of MRI systems or centers to reduce site differences. Alternatively, but not exclusively, data collected from participants who travel between imaging centers (“traveling subjects”) can be used to estimate differences in MRI data across imaging centers in parallel with constructing a large‐scale, multi‐center, multi‐disorder database.^33^, ^44^

By using a novel harmonization method based on traveling subject data,^33^ we have already developed a diagnostic neuroimaging marker of major depressive disorder (MDD), which can generalize to independent datasets acquired from multiple, independent imaging centers.^45^ Thus, it is now becoming technically plausible to investigate neurobiological relationships among multiple psychiatric disorders by applying this generalizable brain network marker of MDD to other psychiatric disorders and *vice versa*.^17^, ^45^ Regrettably, this important activity is currently limited by the paucity of MRI databases including multi‐disorders from multiple centers. There are currently too few of them, as will be reviewed below. Therefore, it is critical to construct a large‐scale, multi‐center, multi‐disorder brain imaging database for psychiatric and neurological disorders.^46^

### The aims of this review

Here we performed a comprehensive review of publicly shared databases of psychiatric disorders worldwide. We also reviewed databases for relevant neurological disorders in aged samples. We did not intend to summarize all the findings generated from those databases. Rather, we created an organized inventory to help clinicians and researchers navigate and interpret findings from the existing databases. We also summarized limitations and problems with such databases to help clinicians understand the necessity for next‐generation databases. We also expected that this review would help advance international data governance for neuroscience.^31^ Various databases have already been established, funded by different initiatives and countries. For this reason, they are often difficult for researchers in other countries to identify and access. Herein, we summarize the current global state of MRI databases for psychiatric and neurological disorders.

We put special emphasis on the following:

1. Significance of multi‐center, multi‐disease databases in developing imaging biomarkers
2. List of MRI databases of psychiatric and neurological disorders
3. Future directions for human brain MRI databases

## Methods

We searched databases or datasets published until February 2022. The steps of the database search are provided below.

**Step 1: Search the datasets for each disease in the following order:**

1. Search for “disease name + MRI” in *Scientific Data*
2. Search by “disease name + MRI” in data repositories such as *OpenNeuro* and *Synapse*
3. Search by “disease name + MRI ‐neuroVault ‐figshare ‐mendeley ‐integbio” using a Google dataset search
4. Search PubMed using the following criteria:
  “(disease name)/diagnostic imaging” (MeSH) AND “Magnetic Resonance Imaging” (MeSH) AND dataset.

If the dataset used there is public and does not appear in 1–3, add it to the list.

- If there are more than 100 hits

Check the top 100 and add them to the list if the dataset is public and does not appear in 1–3.

b If there are 100 or fewer

Check all of them and add them to the list if the dataset is public and does not appear in 1–3.

b If no hits are found.

Described as “N/A.”

*See PubMed MeSH terms for notation of disease name.

Example: depression.

“Depression/diagnostic imaging”[MeSH] AND “Depressive Disorder/diagnostic imaging”[MeSH].

**Step 2: Fill in the following items for each disease in a spreadsheet for the dataset compiled by the above procedure.**

- Search criteria (1–4 described above)
- Database reference Information
- Platform URL
- Data access (fully accessible (open access), application‐based sharing, and collaboration‐based sharing)
- Number of samples
- Contents and format of data
- Number of citations
- Features description of the database

Target datasets were only for human MRI and published datasets that are available directly from websites or for which detailed information on accessing the datasets is available on the websites. Here, we defined MRI dataset as structural MRI, functional MRI, and diffusion MRI. We excluded datasets that are only available *via* email to the corresponding authors of published articles. We summarized items and features of datasets in each psychiatric and neurological disorder. If the number of samples described on portals of the datasets differed from those in journal papers, we adopted the portal site information. If the dataset included multiple disorders, we listed the same dataset for each disorder. We excluded datasets that do not include raw or preprocessed MRI data.

For eating disorders, we used a different searching policy because we did not find any dataset coherent with the above policy. To identify existing open brain MRI database of eating disorders, we searched the following electronic databases: Scientific Data, OpenNeuro, Synapse, Google dataset search, and PubMed. Keywords used for the search were eating disorders, anorexia nervosa, bulimia nervosa, binge‐eating disorder, and MRI. To ensure reliability, four researchers (SH, NN, TN, and MF, co‐authors of this manuscript) carried out the search and identified databases independently. Eligibility criteria for the original studies to be included in the review were as follows: (1) individuals from all age groups or generations were included in the study; (2) a mention about an open brain MRI database for eating disorders; (3) written in English.

We excluded the following datasets:

- Datasets with policy of collaborative‐based sharing in which collaboration is closed within their own consortium.
- ENIGMA in which a collaborative project shares only meta‐data.
- UK Biobank, which is not specific to individual psychiatric/neurological disorder and rather represents a cohort study.
- Human connectome projects where the datasets already released (young adults and lifespan) are not specific to individual psychiatric/neurological disorder and rather represents a cohort study.

All figures were plotted using R (ver. 4.2.0).

## Results

### Overview of worldwide MRI databases of psychiatric and neurological disorders

**Fig. 1:**
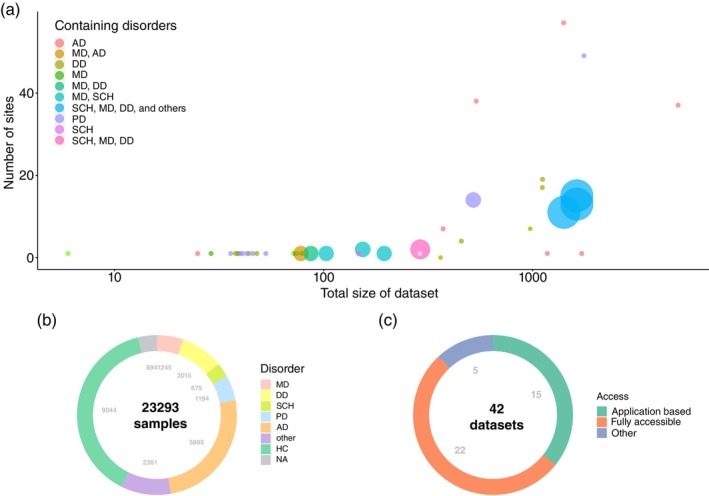
Summary of shared MRI datasets of psychiatric and neurological disorders. (a) The number of sites that collected the data and the total sample size of the datasets. Each marker size indicates the number of disorders included in the dataset. (b) The population of participants pertaining to each disorder. Other information includes patients with disorders other than the six targeted disorders. NA indicates that information about the number of patients and healthy controls is not available (mixed). (c) The access level of the datasets. Other information includes exceptional cases. See Tables 1, 2, 3, 4, 5 of each dataset for details. AD, Alzheimer's disease; DD, developmental disorders; HC, healthy control participants; MD, mood disorders; PD, Parkinson's disease; SCH, schizophrenia.

We systematically surveyed MRI datasets focused on psychiatry and neurological disorders. Figure 1 summarizes shared MRI datasets of psychiatric and selected neurological disorders. We found 43 datasets that fitted the search criteria. We categorized these datasets into six: (1) mood disorders (including MDD and bipolar disorder (BD)), (2) developmental disorders (including autism spectrum disorder (ASD), attention‐deficit hyperactivity disorder (ADHD)), (3) schizophrenia, (4) Parkinson's disease (PD), (5) dementia (including Alzheimer's disease (AD)), and (6) eating disorders.

Figure 1a shows the number of sites that provided data to each dataset and sample sizes of the datasets. Although most datasets include data collected at a single site, several datasets are from multiple centers (57 sites of ADNI is the maximum). Although most datasets consist of a single disorder and healthy individuals, ten datasets include multiple disorders. Figure 1b shows the proportion of participants included in 43 datasets for each disorder. The sample size of neurological disorders, especially AD, is larger than that of psychiatric disorders. Figure 1c shows the proportion of the access level of the datasets: fully accessible (open access to public) (22 projects, number 3, 4, 5, 6, 8, 9, 10, 11, 12, 13, 15, 16, 17, 18, 21, 29, 30, 31, 32, 34, 35, 37) or application‐based sharing (15 projects, number 1, 2, 7, 14, 19, 20, 24, 26, 27, 36, 38, 39, 40, 41, 42) in which application for data usage must be approved. We excluded several datasets with a policy of collaborative‐based sharing in which access was restricted to within their own consortium.

The participant proportion of the datasets are quite different across disorders as shown in Figure 1b. Moreover, features differ across these datasets for the same target disorder.

We plot the proportion of disorders in each dataset (Fig. S1) and provide the information of each dataset for mood disorders (Table 1), developmental disorders (Table 2), schizophrenia (Table 3), PD (Table 4), and AD (Table 5).

**Table 1:** Datasets including patients with mood disorders

	Multi‐disorder Connectivity Dataset (SRPBS‐FC)https://bicr‐resource.atr.jp/srpbsfc/				
1	Search criterion	Data access	Number of samples	Contents	Number of citations
	1	Application‐based sharing	HC: 942, BD:4, MDD:251 ASD: 125, SCH: 146 Dysthymia: 4, OCD: 11, Chronic pain/Back pain: 67, Stroke: 10, Others: 28	Functional‐connectivity matrix, clinical information	19
	This dataset provides the resting state functional connectivity matrix (.mat file) mainly of nine disorders collected at 15 sites with moderately common imaging protocols. For more details, see https://www.nature.com/articles/s41597‐021‐01004‐8.				

	Multidisorder MRI Dataset; restricted (SRPBS‐1600)https://bicr‐resource.atr.jp/srpbs1600/				
2	Search criterion	Data access	Number of samples	Contents	Number of citations
	1	Application‐based sharing	HC: 950, BD: 41, MDD: 255, ASD: 125, SCH: 147, Dysthymia: 4, Chronic pain/Back pain: 67, Stroke: 10, Others: 28	T1w, rs‐fMRI, field map, clinical information	19
	This dataset provides raw MRI data mainly of eight disorders collected in 13 sites with moderately common imaging protocols.				

	Multidisorder MRI Dataset; unrestricted (SRPBS‐open) https://bicr‐resource.atr.jp/srpbsopen/				
3	Search criterion	Data access	Number of samples	Contents	Number of citations
	1	Fully accessible	HC: 791, BD: 41, MDD: 255, ASD: 125, SCH: 147, Dysthymia: 4 Chronic pain/Back pain: 24, Others: 23	T1w, rs‐fMRI, field map, clinical information	19
	This dataset provides raw MRI data mainly of eight disorders collected at 11 sites with moderately common imaging protocols. This is a subset of #2 (SRPBS‐1600) consisting of data from participants who agreed to open access.				

	UCLA Consortium for Neuropsychiatric Phenomics LA5c Studyhttps://openfmri.org/dataset/ds000030/versions/00016				
4	Search criterion	Data access	Number of samples	Contents	Number of citations
	1	Fully accessible	HC: 138, BD: 49, ADHD: 45, SCH: 58	T1w, rs‐fMRI, task‐fMRI, DWI, clinical information	96
	It provides rs‐fMRI, T1w, DWI, and six task‐fMRI (breath‐hold, balloon analog risk, stop signal, task switching, spatial working memory capacity, paired association memory encoding/retrieval) datasets of adult patients with schizophrenia, bipolar disorder, and ADHD. It provides YMRS, BIS‐11, and WAIS. Not all subjects have all task fMRI. BIDS‐like format. It is duplicated in the list of developmental disorders (Table 2) and schizophrenia (Table 3).				

	Alzheimer's Disease versus Bipolar Disorder versus Health Control MRI data and processed resultshttps://doi.org/10.5281/zenodo.3935636				
5	Search criterion	Data access	Number of samples	Contents	Number of citations
	3	Fully accessible	HC: 19, BD: 24, AD: 35	T1w, DWI, clinical information	30
	Data set including subjects aged 50 years and older. It is duplicated in the list of AD (Table 4).				

	Data from Common gray and white matter abnormalities in schizophrenia and bipolar disorderhttps://datadryad.org/stash/dataset/doi:10.5061/dryad.j6q573n9h				
6	Search criterion	Data access	Number of samples	Contents	Number of citations
	3	Fully accessible	HC: 65, BD: 65, SCH: 65	T1w, DWI	7
	This provides the preprocessed T1w and DTI data as well as de‐identified clinical data. Only one nii.gz file per group, per image. MINI was performed (BPRS‐18 and WHODAS 2.0 scores were not disclosed).				

	Brain/MINDS 3D Human Brain Image Datasethttps://dataportal.brainminds.jp/exp‐3775				
7	Search criterion	Data access	Number of samples	Contents	Number of citations
	3	Application‐based sharing	HC: 97, BD: 16, MDD: 21, SCH: 20	T1w	‐
	This dataset includes T1‐weighted images of patients with schizophrenia, those with MDD, and those with BD, as well as of healthy controls. Data set on 20–60‐year‐olds measured at two sites.				

	CANDI Share: Schizophrenia Bulletin 2008https://www.nitrc.org/projects/cs_schizbull08/				
8	Search criterion	Data access	Number of samples	Contents	Number of citations
	3	Fully accessible	HC: 29, BD with psychosis: 19, BD without psychosis: 35, SCH: 20	T1w	86
	A part of CANDI Neuroimaging Access Point (https://www.nitrc.org/projects/candi_share/). This dataset includes preprocessed MRI images (1.5T) and segmentation results of all four diagnostic groups. demographic and behavioral data, and a set of related morphometric resources (static and dynamic atlases). It is duplicated in the list of schizophrenia (Table 3).				

	Neural Processing of Emotional Musical and Nonmusical Stimuli in Depressionhttps://openneuro.org/datasets/ds000171/versions/00001				
9	Search criterion	Data access	Number of samples	Contents	Number of citations
	2	Fully accessible	HC: 20, MDD: 19	T1w, task‐fMRI	‐
	This provides task‐fMRI of 19 individuals with MDD and 20 never‐depressed HC listening to positive and negative emotional musical and nonmusical stimuli. BIDS format.				

	Depression Treatment by Medial Prefrontal Real‐Time fMRI Neurofeedbackhttps://openneuro.org/datasets/ds003770/versions/1.0.0				
10	Search criterion	Data access	Number of samples	Contents	Number of citations
	2	Fully accessible	Mild MDD & Moderate MDD:6	T1w, clinical information task‐fMRI during neurofeedback	‐
	This includes eight sessions of real‐time fMRI neurofeedback targeting the medial prefrontal cortex for six patients with mild and moderate depression. BIDS format.				

	Resting state with closed eyes for patients with depression and healthy participantshttps://openneuro.org/datasets/ds002748/versions/1.0.5				
11	Search criterion	Data access	Number of samples	Contents	Number of citations
	2	Fully accessible	HC: 21, Mild MDD: 51	T1w, rs‐fMRI, clinical information	4
	This provides resting‐state fMRI data of 51 subjects with mild depression and 21 HC. BIDS format.				

	Two sessions of resting state with closed eyes for patients with depression in treatment course (NFB, CBT or No treatment groups)https://openneuro.org/datasets/ds003007/versions/1.0.1				
12	Search criterion	Data access	Number of samples	Contents	Number of citations
	2	Fully accessible	Mild MDD: 29 (2 sessions) – no treatment: 15 – Cognitive behavioral therapy: 8 – Neurofeedback: 6	T1w, rs‐fMRI, clinical information	4
	This dataset provides rs‐fMRI of patients with mild depression. This includes rs‐fMRI data of 2‐month intervals of three groups of patients with cognitive behavioral therapy, fMRI neurofeedback treatment, or without treatment. This dataset and the above dataset are the part of the same study (https://www.hindawi.com/journals/np/2021/8846097/). BIDS format.				

	Cortical myelin measured by the T1w/T2w ratio in individuals with depressive disorders and healthy controls.https://openneuro.org/datasets/ds003653/versions/1.0.0				
13	Search criterion	Data access	Number of samples	Contents	Number of citations
	2	Fully accessible	HC: 47, MDD: 27 Pervasive developmental disorders: 13	T1w, T2w, fieldmaps, clinical information	‐
	This dataset includes T1w, T2w, and spin‐echo field maps of patients with MDD and HC. A more detailed description can be found here https://www.medrxiv.org/content/10.1101/2021.02.25.21252472v2 It is duplicated in the list of developmental disorders (Table 2). BIDS format.				

**Table 2:** Datasets including patients with developmental disorders.

	Multidisorder Connectivity Dataset (SRPBS‐FC) https://bicr‐resource.atr.jp/srpbsfc/				
1	Search criterion	Data access	Number of samples	Contents	Number of citations
	1	Application‐based sharing	HC: 942, BD:4, MDD:251 ASD: 125, SCH: 146 Dysthymia: 4, OCD: 11, Chronic pain/Back pain: 67, Stroke: 10, Others: 28	Functional‐connectivity matrix, clinical information	19
	This is duplicated in the list of mood disorders (Table 1) and schizophrenia (Table 3).				

	Multidisorder MRI Dataset; restricted (SRPBS‐1600) https://bicr‐resource.atr.jp/srpbs1600/				
2	Search criterion	Data access	Number of samples	Contents	Number of citations
	1	Application‐based sharing	HC: 950, BD: 41, MDD: 255, ASD: 125, SCH: 147, Dysthymia: 4, Chronic pain/Back pain: 67, Stroke: 10, Others: 28	T1w, rs‐fMRI, field map, clinical information	19
	This is duplicated in the list of mood disorders (Table 1) and schizophrenia (Table 3).				

	Multidisorder MRI Dataset; unrestricted (SRPBS‐open) https://bicr‐resource.atr.jp/srpbsopen/				
3	Search criterion	Data access	Number of samples	Contents	Number of citations
	1	Fully accessible	HC: 791, BD: 41, MDD: 255, ASD: 125, SCH: 147, Dysthymia: 4 Chronic pain/Back pain: 24, Others: 23	T1w, rs‐fMRI, field map, clinical information	19
	This is duplicated in the list of mood disorders (Table 1) and schizophrenia (Table 3).				

	UCLA Consortium for Neuropsychiatric Phenomics LA5c Study https://openneuro.org/datasets/ds000030/versions/00016				
4	Search criterion	Data access	Number of samples	Contents	Number of citations
	1	Fully accessible	HC: 138, BD: 49, ADHD: 45, SCH: 58	T1w, rs‐fMRI, task‐fMRI, DWI, clinical information	93
	It is duplicated in the list of mood disorders (Table 1) and schizophrenia (Table 3).				

	Autism Brain Imaging Data Exchange I (ABIDE I)				
19	Search criterion	Data access	Number of samples	Contents	Number of citations
	2	Application‐based sharing (must be logged into NITRC)	TD: 573, ASD: 539	T1w, rs‐fMRI, DTI, clinical information	1017
	A multi‐center dataset of children to adults with ASD collected at 17 sites. It provides rs‐fMRI and T1w. Only one site provides DTI. WASI, Handedness, and medication information are available, but not in all centers. Imaging protocols and collection of clinical data are not unified, and data from childhood and adolescence are abundant, while data from adults are limited.				

	Autism Brain Imaging Data Exchange II (ABIDE II) http://fcon_1000.projects.nitrc.org/indi/abide/abide_II.html				
14	Search criterion	Data access	Number of samples	Contents	Number of citations
	1	Application‐based sharing (must be logged into NITRC)	TD: 593, ASD: 521	T1w, rs‐fMRI, DWI, clinical information	185
	A multicenter dataset of ASD from children to adults. It provides rs‐fMRI and T1 w from 18 sites, DWI from five sites, and longitudinal data for two sites. It also provides on ADOS, ADI‐R, SRS, AQ, and Vineland and information of medication. Imaging protocols and clinical data collection are not unified.				

	Moral judgments of intentional and accidental moral violations across Harm and Purity domains https://openneuro.org/datasets/ds000212/versions/1.0.0				
15	Search criterion	Data access	Number of samples	Contents	Number of citations
	2	Fully accessible	TD: 25, ASD: 14	T1w, task‐fMRI	‐
	This provides T1w and task‐fMRI during moral judgment tasks for intentional and unintentional moral violations of adult ASD. BIDS format.				

	Response inhibition and selective attention in adults and children with and without ADHD https://openneuro.org/datasets/ds003500/versions/1.2.0				
16	Search criterion	Data access	Number of samples	Contents	Number of citations
	2	Fully accessible	TD: 26, ADHD: 12	T1w, task‐fMRI	0
	This provides T1w, task‐fMRI (selective attention, response inhibition) dataset of ADHD in children to adults. About half provide longitudinal data. BIDS format.				

	Working Memory and Reward in Children with and without Attention Deficit Hyperactivity Disorder (ADHD) https://openneuro.org/datasets/ds002424/versions/1.2.0				
17	Search criterion	Data access	Number of samples	Contents	Number of citations
	2	Fully accessible	TD: 44, ADHD: 35	T1w, task‐fMRI, clinical information	‐
	This provides T1w, task‐fMRI (visuospatial working memory) dataset of children with ADHD. About half provide longitudinal data. It provides performance on cognitive tasks such as WASI, ADHD‐RS, and CPT. BIDS format.				

	Contrast Response Functions https://openneuro.org/datasets/ds002522/versions/1.0.0				
18	Search criterion	Data access	Number of samples	Contents	Number of citations
	2	Fully accessible	TD: 24, ASD: 24	T1w, task‐fMRI	6
	This provides T1w and task‐fMRI (grating orientation) of adult with ASD. BIDS format.				

	ADHD‐200 http://fcon_1000.projects.nitrc.org/indi/adhd200/				
20	Search criterion	Data access	Number of samples	Contents	Number of citations
	2	Application‐based sharing (must be logged into NITRC)	TD: 585 ADHD: 362 Diagnosis unavailable: 26	T1w, rs‐fMRI, clinical information	86
	A multicenter dataset of rs‐fMRI and T1w of children with ADHD collected at eight sites. Symptom assessments, such as ADHD‐RS and CPRS‐LV, and IQ and medication status, such as WASI and WISC, are provided. The imaging protocol is not unified.				

	Biomarker Research in ADHD: the Impact of Nutrition (BRAIN) – functional MRI data set https://easy.dans.knaw.nl/ui/datasets/id/easy‐dataset:227983				
21	Search criterion	Data access	Number of samples	Contents	Number of citations
	3	Fully accessible	TD: 26, ADHD: 53	T1w, task‐fMRI, clinical information	‐
	This provides T1w, task‐fMRI (Stop‐signal, Flanker) dataset of children with ADHD before and after a five‐week few‐foods diet. ADHD‐RS scores are used for behavioral assessment. Medication status and IQ scores prior to study initiation are provided. BIDS format.				

	Longitudinal MRI Study of Infants at Risk for Autism https://nda.nih.gov/edit_collection.html?id=19				
22	Search criterion	Data access	Number of samples	Contents	Number of citations
	3	Other (must be logged into NDA, submit data access request, and reviewed by the NDA Data Access Committee)	ASD: 456*	T1w, rs‐fMRI, DWI, clinical information	‐
	A dataset provided by IBIS Network (https://ibis‐network.com/). T1w, rs‐fMRI data sets at 6, 12, and 24 months in young children at high familial risk for ASD. In addition to ADOS and RBS‐R, several infant clinical assessments are available. *Details are unknown because they cannot be accessed.				

	Biological and Information Processing Mechanisms Underlying Autism https://nda.nih.gov/edit_collection.html?id=6				
23	Search criterion	Data access	Number of samples	Contents	Number of citations
	3	Other (must be logged into NDA, submit data access request, and reviewed by the NDA Data Access Committee)	ASD: 75*	fMRI, DWI, Structural, clinical information	‐
	Data set of children to adults with ASD. There are only 75 samples, but about 200 to 300 images have been provided for ADOS symptom evaluation and cognitive function tests. *Details are unknown because they cannot be accessed.				

	Adolescents with autism show typical fMRI repetition suppression, but atypical surprise response. https://data.donders.ru.nl/collections/di/dccn/DSC_3018026.01_044?0				
24	Search criterion	Data access	Number of samples	Contents	Number of citations
	3	Application‐based sharing	TD: 22, ASD: 22	T1w, task‐fMRI	‐
	A dataset that measures brain activity when visual stimuli about objects were presented.				

	Biomarkers of Autism at 12 months: From Brain Overgrowth to Genes https://nda.nih.gov/edit_collection.html?id=9				
25	Search criterion	Data access	Number of samples	Contents	Number of citations
	4	Other (must be logged into NDA, submit data access request, and reviewed by the NDA Data Access Committee)	ASD: 363*	T1w, rs‐fMRI, task‐fMRI, DWI, clinical information	‐
	Data set of 12–36‐month‐old infants focused on the development of ASD. It provides rs‐fMRI, task‐fMRI, genome analysis data and clinical evaluations such as ADOS, though not for everyone. *Details are unknown because they cannot be accessed.				

	Cortical myelin measured by the T1w/T2w ratio in individuals with depressive disorders and healthy controls. https://openneuro.org/datasets/ds003653/versions/1.0.0				
13	Search criterion	Data access	Number of samples	Contents	Number of citations
	2	Fully accessible	HC: 47, MDD: 27 Pervasive developmental disorders: 13	T1w, T2w, fieldmaps, clinical information	‐
	This is duplicated in the list of mood disorders (Table 1).				

**Table 3:** Datasets including patients with schizophrenia.

	Multidisorder Connectivity Dataset (SRPBS‐FC) https://bicr‐resource.atr.jp/srpbsfc/				
1	Search criterion	Data access	Number of samples	Contents	Number of citations
	1	Application‐based sharing	HC: 942, BD:4, MDD:251 ASD: 125, SCH: 146 Dysthymia: 4, OCD: 11, Chronic pain/Back pain: 67, Stroke: 10, Others: 28	Functional‐connectivity matrix, clinical information	19
	This is duplicated in the list of mood disorders (Table 1) and developmental disorders (Table 2).				

	Multidisorder MRI Dataset; restricted (SRPBS‐1600) https://bicr‐resource.atr.jp/srpbs1600/				
2	Search criterion	Data access	Number of samples	Contents	Number of citations
	1	Application‐based sharing	HC: 950, BD: 41, MDD: 255, ASD: 125, SCH: 147, Dysthymia: 4, Chronic pain/Back pain: 67, Stroke: 10, Others: 28	T1w, rs‐fMRI, field map, clinical information	19
	This is duplicated in the list of mood disorders (Table 1) and developmental disorders (Table 2).				

	Multidisorder MRI Dataset; unrestricted (SRPBS‐open) https://bicr‐resource.atr.jp/srpbsopen/				
3	Search criterion	Data access	Number of samples	Contents	Number of citations
	1	Fully accessible	HC: 791, BD: 41, MDD: 255, ASD: 125, SCH: 147, Dysthymia: 4 Chronic pain/Back pain: 24, Others: 23	T1w, rs‐fMRI, field map, clinical information	19
	This is duplicated in the list of mood disorders (Table 1) and developmental disorders (Table 2).				

	UCLA Consortium for Neuropsychiatric Phenomics LA5c Study https://openneuro.org/datasets/ds000030/versions/00016				
4	Search criterion	Data access	Number of samples	Contents	Number of citations
	1	Fully accessible	HC: 138, BD: 49, ADHD: 45, SCH: 58	T1w, rs‐fMRI, task‐fMRI, DWI, clinical information	96
	This is duplicated in the list of mood disorders (Table 1) and developmental disorders (Table 2).				

	Data from Common gray and white matter abnormalities in schizophrenia and bipolar disorder https://datadryad.org/stash/dataset/doi:10.5061/dryad.j6q573n9h				
6	Search criterion	Data access	Number of samples	Contents	Number of citations
	3	Fully accessible	HC: 65, BD: 65, SCH: 65	T1w, DWI	7
	This is duplicated in the list of mood disorders (Table 1).				

	Brain/MINDS 3D Human Brain Image Dataset https://dataportal.brainminds.jp/exp‐3775				
7	Search criterion	Data access	Number of samples	Contents	Number of citations
	3	Application‐based sharing	HC: 97, BD: 16, MDD: 21, SCH: 20	T1w	‐
	This is duplicated in the list of mood disorders (Table 1).				

	CANDI Share: Schizophrenia Bulletin 2008 https://www.nitrc.org/projects/cs_schizbull08/				
8	Search criterion	Data access	Number of samples	Contents	Number of citations
	3	Fully accessible	HC: 29, BD with Psychosis: 19, BD without Psychosis: 35, SCH: 20	T1w	86
	This is duplicated in the list of mood disorders (Table 1).				

	The Center for Biomedical Research Excellence (COBRE) http://fcon_1000.projects.nitrc.org/indi/retro/cobre.html				
26	Search criterion	Data access	Number of samples	Contents	Number of citations
	3	Application‐based sharing (must be logged into NITRC)	HC: 75, SCH: 72	T1w, fMRI, DWI	51
	Part of the 1000 Functional Connections Project (NITRC). This provides MRI data from 72 patients with schizophrenia and 75 HC. Registration for an account with NITRC must be needed.				

**Table 4:** Datasets including patients with Parkinson's disease.

	Parkinson's Progression Markers Initiative (PPMI) https://www.ppmi‐info.org/				
27	Search criterion	Data access	Number of samples	Contents	Number of citations
	1	Application‐based sharing	HC: 237, PD: 902, Prodromal: 619	T1w, T2‐FLAIR, fMRI, DWI, clinical information	641
	Multimodal MRI datasets for untreated PD. PET and SPECT (DAT scan) data are also available. This also stores biological samples (CSF, serum, and plasma), genetic information (DNA, RNA), and clinical information. Not only cross‐sectional data, but also longitudinal data exist, and longitudinal data exist from prodromal PD to PD.				

	Oxford Parkinson's Disease Centre Discovery Cohort (OPDC Discovery) https://portal.dementiasplatform.uk/				
28	Search criterion	Data access	Number of samples	Contents	Number of citations
	1	Others*	HC: 68, early iPD:119, PD with pathogenic mutations of the leucine‐rich repeat kinase 2 or GBA: 15, Risk of PD:87	T1w, T2‐FLAIR, rs‐fMRI, DWI, SWI , NM‐MRI clinical information	1
	This is a multimodal MRI data set for untreated PD. Detailed data from basic subject information include family history, motor function, cognitive function, mental health, function of daily living, and daily living (Drinking history, smoking history, etc.). In addition to cross‐sectional data, there is also profile data. * Collaboration‐based sharing, but the data are provided within a virtual desktop. The applicants can analyze the data on the virtual desktop.				

	ANT: Healthy aging and Parkinson's disease https://openneuro.org/datasets/ds001907/versions/2.0.3				
29	Search criterion	Data access	Number of samples	Contents	Number of citations
	2	Fully accessible	HC: 21, PD: 25	T1w, task‐fMRI clinical information	‐
	Data set of T1w and fMRI using the attention network test. Detailed neuropsychological tests are also measured. BIDS format.				

	High‐quality diffusion‐weighted imaging of Parkinson's disease https://www.nitrc.org/projects/parktdi				
30	Search criterion	Data access	Number of samples	Contents	Number of citations
	2	Fully accessible	HC: 26, PD: 27	DWI, TDI, clinical information	26
	This dataset of DWI with motion correction was made to carry out the connectome analysis by tract density imaging. Evaluation of motor symptoms and neuropsychological tests are also performed.				

	White matter alterations in Parkinson's disease with normal cognition precede gray matter atrophy https://datadryad.org/stash/dataset/doi:10.5061/dryad.b4q8k				
31	Search criterion	Data access	Number of samples	Contents	Number of citations
	3	Fully accessible	HC: 21, PD: 20	T1w, DWI, clinical information	51
	Datasets for analysis of gray matter, white matter volume and density, and white matter diffusion anisotropy. Multidomain cognitive testing is being done.				

	Cortical Oscillatory Dysfunction in Parkinson Disease During Movement Activation and Inhibition https://doi.gin.g‐node.org/10.12751/g‐node.nb35ss/				
32	Search criterion	Data access	Number of samples	Contents	Number of citations
	3	Fully accessible	HC: 18, PD: 18	T1w, MEG	‐
	T1w MRI data and MEG data measured in response initiation and inhibition tasks of control subjects and subjects with PD.				

	Ontario Neurodegenerative Disease Research Initiative https://www.braincode.ca/content/controlled‐data‐releases#dr007				
33	Search criterion	Data access	Number of samples	Contents	Number of citations
	5	Other*	AD/ MCI: 126, PD: 140 FTD: 53, CVD:161, ALS:40	Regional diffusivity using T1w, T2*, T2‐FLAIR, rs‐fMRI, DWI, Structural volumes, WMH, gradient echo, clinical information	56
	Multi‐Neurological disease cohort available for requests on the web. *This dataset is accessible only on their provided workspace. It is duplicated in the list of AD (Table 5).				

	NEUROCON rs‐fMRI study http://fcon_1000.projects.nitrc.org/indi/retro/parkinsons.html				
34	Search criterion	Data access	Number of samples	Contents	Number of citations
	5	Fully accessible	HC: 16, PD: 27	T1w, rs‐fMRI	31
	This dataset includes T1w and resting‐state MRI (1.5T) of 27 PD patients and 16 age‐matched HC.				

	Tao Wu http://fcon_1000.projects.nitrc.org/indi/retro/parkinsons.html				
35	Search criterion	Data access	Number of samples	Contents	Number of citations
	5	Fully accessible	HC: 20, PD: 20	T1w, rs‐fMRI	‐
	This dataset includes T1w and resting‐state MRI (3T) of 20 PD patients and 20 age‐matched HC.				

**Table 5:** Datasets including patients with Alzheimer's disease.

	Alzheimer's Disease *versus* Bipolar Disorder *versus* Health Control MRI data and processed results https://doi.org/10.5281/zenodo.3935636				
5	Search criterion	Data access	Number of samples	Contents	Number of citations
	3	Fully accessible	HC: 19, BD: 24, AD: 35	T1w, DWI, clinical information	30
	Discovering AD and BD white matter effects building computer aided diagnostic systems on brain diffusion tensor imaging features. It is duplicated in the list of mood disorders (Table 1).				

	Ontario Neurodegenerative Disease Research Initiativehttps://www.braincode.ca/content/controlled‐data‐releases#ond01				
33	Search criterion	Data access	Number of samples	Contents	Number of citations
	5	Other*	AD/MCI: 126, PD: 140, FTD: 53, CVD:161, ALS:40	Regional diffusivity using T1w, T2*, T2‐FLAIR, rs‐fMRI, DWI, Structural volumes, WMH, gradient echo, clinical information	56
	Multi‐Neurological disease cohort available for requests on the web. *This dataset is accessible only on their provided workspace. It is duplicated in the list of PD (Table 5).				

	Alzheimer Disease Neuroimaging Initiative (ADNI)‐1/2 http://adni.loni.usc.edu/				
36	Search criterion	Data access	Number of samples	Contents	Number of citations
	1	Application‐based sharing	ADNI phase 1 AD: 188, MCI: 392, Cognitively normal subjects: 226 ADNI phase 2 (Added‐on ADNI phase1) Elderly HC: +150, Early MCI: +300, Late MCI: +150	MRI: T1 3D, T2*GRE, hippocampal T2, rs‐fMRI, DWI, FLAIR, ASL, PET: FDG, [(18)F]florbetapir (1600 <) clinical information	67
	These datasets provide multicenter, AD‐only prospective cohort datasets with multi‐modal dataset including neuroimaging, clinical information, and biological samples.				

	Open Access Series of Imaging Studies (OASIS)‐1/2/3 https://www.oasis‐brains.org/				
37	Search criterion	Data access	Number of samples	Contents	Number of citations
	1	Fully accessible	OASIS‐1 Nondemented: 316, Demented: 100 OASIS‐2 Nondemented: 72, AD/demented: 115, Change to demented from nondemented: 14 OASIS‐3 Nondemented: 609, AD/demented: 489	OASIS‐1 MRI: T1w OASIS‐2 MRI: T1w OASIS‐3 MRI: T1w, T2w, rs‐fMRI, DWI, FLAIR, TSE, SWI, ASL, fieldmaps, PET: FDG, F18‐Florbetapir (1600 <) clinical information	1242
	These datasets provide multi‐modal dataset including neuroimaging, clinical, cognitive, and biomarker dataset for HC and AD. They are hosted by central.xnat.org.				

	National Alzheimer's Coordinating Center https://naccdata.org/				
38	Search criterion	Data access	Number of samples	Contents	Number of citations
	2	Application‐based sharing	Normal cognition: 1978, Impaired, not MCI: 467, MCI: 806, Dementia: 1703	MRI: T1,T2,DWI,FLAIR PET: FDG, [(18)F]florbetapir (600 <), tau clinical information	550
	AD datasets collected from 37 Alzheimer's Disease Research Centers (ADRCs) across 26 states. Both DICOM and NIfTI formats are available.				

	PennMTLAtlashttps://openneuro.org/datasets/ds003052/versions/1.1.0				
39	Search criterion	Data access	Number of samples	Contents	Number of citations
	2	Fully accessible	AD: 25	T2w	1
	This dataset includes raw T2w, MRI images with intensity clipped, and preprocessed data focused on the medial temporal lobe atrophy due to AD pathology using *ex vivo* MRI and histopathology. BIDS format.				

	J‐ADNI https://humandbs.biosciencedbc.jp/en/hum0043‐v1				
40	Search criterion	Data access	Number of samples	Contents	Number of citations
	3	Application‐based sharing	Elderly HC: 154, AD: 149, MCI: 234	MRI: T2, MP‐RAGE, B1 PET: FDG (150~), 18F‐flutemetamol (150~) clinical information	25
	The dataset includes structural MRI analysis, FDG and amyloid PET, CSF sampling, apoE genotyping, combined with a set of clinical and psychometric tests that were prepared to achieve the highest compatibility to those used in US‐ADNI.				

	The Washington Heights–Inwood Columbia Aging Project https://dss.niagads.org/studies/sa000007/ Data request portal site: https://cumc.co1.qualtrics.com/jfe/form/SV_6x5rRy14B6vpoqN				
41	Search criterion	Data access	Number of samples	Contents	Number of citations
	3	Application‐based sharing	WHICAP‐1 Total: 690, AD: 34, MCI: 122 WHICAP‐2 Total: 485, AD: 8, MCI: 77	MRI: T1w, FLAIR PET: 18F‐Florbetaben (100~) clinical information	68
	These datasets are collected by a prospective, community‐based longitudinal study of aging and dementia in northern Manhattan. Forty‐two participants with MRI data died after the MRI and follow‐up data had been acquired and underwent autopsy.				

	The Italian Alzheimer's Disease Neuroimaging Initiative (I‐ADNI)https://www.gaaindata.org/partner/I‐ADNI				
42	Search criterion	Data access	Number of samples	Contents	Number of citations
	3	Application‐based sharing	AD: 253, MCI: 92, memory impairment: 12, Frontotemporal dementia: 16	T1w, T2w	63
	This dataset consists of patients with subjective memory impairment, mild cognitive impairment, AD and frontotemporal dementia enrolled in seven Italian centers.				

### Features of datasets in each disorder

#### Mood disorders (Table 1)

We found 13 datasets with 1245 samples from patients with mood disorders, diagnosed as MDD or BD. Five datasets include MDD only, four datasets include BD only, and four datasets include both. Most of the data were collected in Japan, followed by the USA and Russia. Twelve datasets include structural MRI data only (project number 2–13), seven datasets provide structural and functional MRI data (project number 2–4, 9–12), and one provides functional connectivity (FC) matrix calculated from resting‐state functional MRI data (project number 1). Three datasets also provide diffusion MRI data (project number 4–6).

The biggest datasets for MDD are the Multi‐disorder Connectivity Dataset (*n* = 251, SRPBS‐FC, project number 1), the Multi‐disorder MRI Dataset with application‐based sharing (*n* = 255, SRPBS‐1600, project number 2), and fully accessible SRPBS‐open (*n* = 255, project number 3). The original source of these three datasets is the same, but different participants are included depending on their approved informed consent. SRPBS‐FC includes processed functional connectivity data of resting‐state fMRI for participants who agreed with application‐based sharing. SRPBS‐1600 includes T1‐weighted (T1w) and resting state fMRI (rs‐fMRI) for participants who agreed with application‐based sharing. SRPBS‐open includes T1w and rs‐fMRI data for participants who agreed with open access to public. The latter two datasets include the same MDD patients' data because all patients with MDD included in SRPBS‐1600 agreed with open access. In other datasets, the number of patients with MDD is smaller than about 50. Existing MRI datasets from MDD are relatively larger than other psychiatry disorders but primarily cross‐sectional. Longitudinal MRI datasets from patients who received treatments are limited. Project number 12 includes two rs‐fMRI datasets with 2‐month intervals from three groups of patients who underwent cognitive behavioral therapy (*n* = 8), fMRI neurofeedback treatment (*n* = 6), or no treatment (*n* = 15). The Global ECT‐MRI Research Collaboration (https://helse-bergen.no/en/avdelinger/psykisk-helsevern/forskingsavdelinga-divisjon-psykisk-helsevern/gemric-the-global-ect-mri-research-collaboration) has collected longitudinal MRI datasets from patients with MDD or BP who received electroconvulsive therapies (however, we excluded this dataset because the collaboration is limited to the consortium). For datasets with MDD, we found different types of patients such as patients with mild MDD (project number 10, 11, and 12) or moderate MDD (project number 10).

The biggest datasets from patients with BD are Data from Common gray and white matter abnormalities in schizophrenia and BD (*n* = 65, project number 6). This dataset provides structural MRI data (T1 weighted MRI (T1w) and diffusion weighted imaging (DWI)), but not functional MRI data. Some datasets from patients with BD derive from studies differentiating late‐onset BP from AD (project number 5) and BD from schizophrenia (project number 6). Only three datasets (project number 2, 3 and 4) provide functional MRI data. Datasets with longitudinal case series were not found.

#### Developmental disorders (Table 2)

We found 17 datasets with 2015 samples from patients with developmental disorders including ASD or ADHD. Eleven datasets include patients with ASD, five datasets include patients with ADHD, and one dataset include pervasive developmental disorders (project number 13). Most of the data were collected in the United States, followed by Japan and the Netherlands. Sixteen datasets include structural MRI data, 15 datasets provide both structural and functional MRI data (project number 2–4,14–25), and one provides FC matrix data (project number 1). Five datasets also provide diffusion MRI data (project number 4, 14, 22, 23, and 25).

The biggest datasets from patients with ASD are the Autism Brain Imaging Data Exchange (ABIDE I, *n* = 539, project number 19; ABIDE II, *n* = 521, project number 14). The biggest dataset from patients with ADHD is ADHD‐200 (*n* = 362, project number 20). The number of patients with ASD included in the datasets is larger than other psychiatry disorders. Most of the existing open MRI datasets from developmental disorders are cross‐sectional, and they focus on data from childhood to adolescence such as in the ABIDE (project number 14 and 19) and the ADHD‐200 (project number 20). MRI data from adults with developmental disorders and longitudinal MRI data are limited. The Multi‐disorder Connectivity and MRI datasets provide cross‐sectional MRI data from adults with developmental disorders (*n* = 125 for ASD, project number 1–3). An ongoing project, Healthy Brain Network (HBN, http://fcon_1000.projects.nitrc.org/indi/cmi_healthy_brain_network/) is a recently‐launched project that has collected multimodal MRI data, including rs‐fMRI, task‐fMRI (during movie‐watching), T1w, T2‐weighted images (T2w), and DWI, from 10,000 participants, including multiple neurodevelopmental disorders. We excluded this dataset because information of the number of patients was not yet available on the project web site nor the data descriptors paper.^47^

A feature of the datasets for developmental disorders is that most of the existing datasets provide functional data (16/17). Only project number 13, focusing on cortical myelin, provides only structural data.

#### Schizophrenia (Table 3)

We found eight datasets with 675 samples from patients with schizophrenia. Most of the data were collected in the United States, followed by Australia and Japan. Eight datasets include structural MRI data, four datasets provide structural and functional MRI data (project number 2–4, 26), and one provides FC matrix data (project number 1). Four datasets also provide diffusion MRI data (project number 4, 6 and 26).

The biggest datasets for datasets from patients with schizophrenia are the Multi‐disorder Connectivity Dataset (*n* = 146, SRPBS‐FC, project number 1), the Multi‐disorder MRI Dataset with application‐based sharing (*n* = 147, SRPBS‐1600, project number 2), and fully accessible SRPBS‐open (*n* = 147, project number 3), as well as MDD datasets. The number of participants with schizophrenia (spectrum disorder) does not exceed 200. None of the databases explicitly include longitudinal data. One aspect of datasets from patients with schizophrenia is that functional MRI data are limited. The Multidisorder Connectivity and MRI Datasets (project number 1–3), UCLA Consortium for Neuropsychiatric Phenomics LA5c Study (project number 4), and The Center for Biomedical Research Excellence (COBRE) provide functional MRI data.

Another feature is that the listed datasets vary in the inclusion criteria. Some include patients with schizophrenia (project number 6, 7 and 26) while others include both schizoaffective disorder and schizophrenia (project number 4), or schizophrenia spectrum disorder (project number 1–3, and 8), possibly based on the interest of the cross‐disorder study.

#### PD (Table 4)

We found nine datasets with 1194 samples from patients with PD. The data were collected in 15 countries, including Austria, Belgium, Canada, China, Czech, France, Germany, Greece, Israel, Netherlands, Norway, Romania, Spain, UK, and USA. Eight datasets include structural MRI data, and six datasets provide structural and functional MRI data (project number 27–29, 33–35). Five datasets provide diffusion MRI data (project number 27, 28, 30, 31, and 33). One dataset provides MEG data (project number 32).

The first notable feature of the datasets from patients with PD is that multi‐center cohorts have been established. Parkinson Progression Marker Initiative (PPMI, project number 27) and Oxford Parkinson's Disease Center (OPDC) Discovery (project number 28) are both large‐scale databases based on multi‐center cohorts that are designed to reveal the underlying pathological processes of PD and its prodromal state. Notably, these two cohorts use the standardized protocol for longitudinal collection of clinical information including non‐motor symptoms such as depression, multimodal imaging data, biospecimens (blood and cerebrospinal fluid), and genetic information.

The second notable feature is that multiple types of MRI such as T1w, fluid‐attenuated inversion recovery (FLAIR), neuromelanin‐sensitive MRI, T2w, susceptibility‐weighted MRI, DWI, and rs‐fMRI are available. The datasets also include positron emission tomography (PET) scans with vesicular monoamine transporter 2, fluorodeoxyglucose tracers, and single‐photon emission computed tomography (SPECT) with dopamine transporter tracers.

The other feature is the availability of longitudinal datasets. The PPMI collects data at baseline, and every half ear later after the entry. The OPDC Discovery collects data at baseline, and at 1.5, 3, and 5 years after the entry.

#### Dementia (Table 5)

We found 10 datasets with 5865 samples from patients with AD. Most of the data were collected in the United States, followed by Canada, Japan and Italy. All datasets include structural MRI data, and three datasets provide both structural and functional MRI data (project number 34, 37, and 38). Five datasets provide diffusion MRI data (project number 5, 33, 36–38) and five datasets provide PET data (project number 36–38, 40, 41).

Databases that collected multimodal imaging and clinical data for AD are categorized into two types based on the target population: the disease cohort and the risk cohort. The risk cohort enrolls, for example, family members who carry genetic risks for developing AD, but the datasets are not publicly open to our knowledge. The disease cohort enrolls clinically diagnosed patients with AD, other dementia‐related disorders, and healthy aged people (project number 36–42 in Table 1). In the disease cohort, most patients are diagnosed with AD or mild cognitive impairment (MCI). Several databases also enrolled patients with other diagnoses such as DLB, frontotemporal dementia, vascular dementia, or PD‐related disorders. The imaging datasets collected structural MRI only (project numbers 38, 5, 40, 41, and 42) or multimodal data including rs‐fMRI (project numbers 36, 37, and 12), and amyloid or tau PET (except for the project number 5). Many projects collected biospecimens such as cerebrospinal fluid to examine biomarkers including amyloid‐β 40/42, phosphorylated tau, total tau, or neurofilament light chain (project numbers 36, 38, 40, 41, and 42). About half of the databases collected genetic information such as lipoprotein ApoE allele (project numbers 36, 38, 40, and 41).

We are unaware of any existing MRI databases for AD that collected autopsied brain tissues. The main scope of the projects above is to investigate changes in brain structure and functional networks (as measured by MRI) that parallel progression in amyloidopathy and/or tauopathy (measured by PET and fluid biomarkers) underlying AD. The rationale behind this policy is based on the strongly held idea that AD is caused by pathological proteins (amyloid‐β and tau) evidenced by countless postmortem pathological and genetic studies.

Notably, the Ontario Neurodegenerative Disease Research Initiative (ONDRI; https://ondri.ca; project number 33) provides a database of multiple neurodegenerative diseases. In addition to data from 140 patients with PD, this cohort also offers data from 126 patients with AD or MCI, 40 patients with amyotrophic lateral sclerosis, 53 patients with frontotemporal dementia, 161 patients with cerebrovascular disease, and patients' healthy spouses. Data collection of ONDRI has been standardized and made public. ONDRI uses the Brain‐CODE (https://www.braincode.ca/) as a data platform to handle MRIs (T1w and T2w, FLAIR, DWI, and rs‐fMRI) and other data types.

#### Eating disorders

We did not find any open brain MRI datasets of eating disorders. Please see the Methods for searching policies.

## Discussion

We surveyed MRI datasets focused on psychiatric and related neurological disorders and found 43 datasets that were either fully accessible (open access to public), application‐based sharing, or collaborative‐based sharing. These datasets collected data from patients with mood disorders (MDD and BD), developmental disorders (ASD and ADHD), schizophrenia, PD, dementia (including AD) and healthy controls. Ten datasets included two or more types of disorders, and the rest included a single type of disorder. Almost all datasets provided T1w, and many datasets included additional multimodal data such as rs‐fMRI or DWI.

The datasets for patients with neurological disorders were larger than for those with psychiatric disorders; datasets for AD were the largest. One possible explanation is the use of neuroimaging data as biomarkers. MRI (especially structural MRI) are commonly used as biomarkers for neurological diseases because of the evidence of association between changes in structure and symptoms, and thus MRI databases have been established. On the other hand, MRI biomarkers for psychiatric disorders are not as established as those for neurological disorders. Although biomarkers using resting‐state fMRI have been shown to be effective,^45^ there are several challenges to sharing fMRI datasets. For example, more stringent measurement conditions and specific equipment for fMRI than for structural MRI limit the number of institutions that can acquire such data, and the greater volume of data required to analyze fMRI results in larger data sizes.

### Current status and future directions for disordered human brain MRI databases

We encountered difficulties in finding basic information such as the number of participants in the datasets. Except for OpenNeuro, information about the datasets was described in an inconsistent manner. It will be crucial in the future for researchers or initiatives that publish datasets to provide users with all necessary descriptive information on the first page of the portal site. For example, all datasets should provide DOIs to allow for clear and unambiguous identification that meets global standards. In performing this review, we realized that one of the biggest issues with existing MRI datasets is the lack of a standard format for dataset description.

Based on these observations, we hereby propose characteristics an ideal portal should have for an MRI database.

#### Standardized format of web site

A portal website with standardized format of the database meta‐data greatly enhances searchability. For example, the IBI data sharing working group recommends schema.org (https://schema.org), an established set of schemas for structured data markup on web pages. The data portal site of the Strategic International Brain Science Research Promotion Program (Brain/MINDS), an IBI member project in Japan, adopts schema.org (https://dataportal.brainminds.jp/). The Brain Imaging Data Structure (BIDS)^48^ is one of the fastest growing standards for neuroimaging data and it is being expanded to other data modalities.^49^ BIDS is not only compatible with OpenNeuro but also with major internationally accessible data analysis platforms such as brainlife.io.^50^

#### Clear descriptions of data access and usage

An ideal data portal should clearly state how the data can be used. The type and range of data availability, the requirements for its use, and the application process must be clearly indicated to applicants.

#### Sustainability of database

The database should be sustained and with clarity for who is responsible for maintenance. The contents must be updated appropriately, such as when adding new data or modifying the data, so that transparent records of changes are kept and reflected in the meta data. If it is difficult to manage or maintain the database upon completion of research funding, it is necessary to take measures such as moving datasets to public repositories.

#### Clear contact details for inquiry

The contact address should be clearly listed on the landing page to accept queries. Applicants should be able to get answers within a reasonable time range.

#### Cross‐cohort queries

When collecting multiple datasets, it is also important to be able to search for datasets with the same concept easily. To perform concept‐based searches, datasets need to be annotated with meta data that enables cross‐cohort searches.^51^ Efforts to enable queries across neuroscience resources, such as the Neuroscience Information Framework (NIF; https://neuinfo.org), would also be useful.

### Across the life‐stage, across the disease categories and across the borders

The brain is the most complicated organ in the human body and is the most difficult to physically examine. Nevertheless, the development and spread of MRI technology has now reached the point where more than a hundred thousand healthy brain MRI scans have been acquired and analyzed, yielding a standard developmental trajectory of the brain across the life stages‐‐from neonates and to centenarians.^52^ Such MRI‐based information can serve as a chart to navigate and discover markers of neuro‐psychiatric disorders spanning the traditional disease categories. In the Strategic International Brain Science Research Promotion Program (Brain/MINDS Beyond, BMB for short, https://brainminds-beyond.jp), an IBI member project in Japan, psychiatrists, neurologists, and neuroscientists are collaborating to find disease markers over the different life stages and traditional disease categories. This approach is even more ambitious than the RDoC project, in which the inference is limited to psychiatric disorders. By focusing on various brain disorders across the lifespan, we aim to identify common and disease‐specific features of psychiatric and neurological disorders. For example, the neural features found in certain psychiatric disorders may be shared across multiple disorders.^53^, ^54^, ^55^, ^56^, ^57^ Such non‐specificity would suggest that neural alterations reported in psychiatric disorders, such as volume changes in subcortical structures in schizophrenia^57^, ^58^ might also occur at different stages in healthy aging and development. Investigating brain structure and function related to neurological and psychiatric disorders across the lifespan may result in the development of more robust disease markers. For example, dementia is seen by both neurologists and psychiatrists in Japan. Even in neurology, there is a growing notion that sticking to traditional disease categories, e.g., AD and PD, may prevent finding true markers of brain disorders^59^ because overlap of AD and PD/DLB pathology is much more common than previously thought. Common in dementia in an aged population are behavioral and psychological symptoms (BPSD) including psychosis, depression, anxiety, anhedonia, and impulsive behaviors. A milder form of these symptoms may even precede the overt onset of AD and PD/DLB. To find the differences and similarities in the neural circuits underlying depressive symptoms across MDD and PD will greatly advance our knowledge about mood and mood disorders. Therefore, we propose that future MRI databases should include data spanning across not only lifespan but also diagnostic categories and clinical departments in hospitals.

We excluded several datasets from our search. For example, the Human Connectome Project (https://www.humanconnectome.org) is a large national project that is part of the Brain Initiative in the U.S. Uniform imaging protocols and pre‐processing methods, as well as large datasets, have been published and continue to make significant contributions to the advancement of neuroscience. One such project is Connectomes Related to Human Diseases (https://www.humanconnectome.org/disease-studies). This is a series of disease‐specific, multi‐center projects, and data are now available for several diseases, including anxiety disorders and early psychosis. Additional datasets are expected to be released in the future.

### An ongoing MRI database project in Japan

The BMB drives the first multicenter study in Japan with unified protocols for MRI measurements and data analyses, focusing on both psychiatric and neurological disorders across the human lifespan.^44^ The goal of the project is to develop imaging biomarkers for psychiatric and neurological disorders in order to classify disease subtypes and to identify comorbidity between diseases, to illuminate pathophysiology, to determine treatment efficacy, and to develop novel therapies. Data from more than 6000 samples from patients with various psychiatric disorders, neurological disorders, and control healthy participants have been collected by March 2024. These data are to be released, at least in part, not too long after the end of the project. Notably, we will publish the first open brain MRI database of eating disorders in the world, including cross‐sectional and longitudinal brain MRI datasets.^60^ The BMB project plans to establish multi‐level harmonization consisting of (1) harmonized MRI protocols, (2) harmonized preprocessing and preliminary data, (3) uniformed high‐quality Quality Controls, and (4) statistical harmonization using traveling subject data. We will establish a database site based on a standardized format of database meta‐data, such as schema.org (https://schema.org), an established set of schemas for structured data markup on web pages and recommended by the IBI data sharing working group.

### Limitations

It is possible that some relevant MRI databases were not included in this study. For example, the NIH Data Archive (NDA), a large data repository provided by the US National Institutes of Health, requires a user account to access the data, and detailed information on data use and download could not be obtained without logging into an account. As we have discussed in this review, there are also many inconsistencies in how MRI datasets are made available, and the detail and accessibility of relevant meta data, as well as where and how the datasets have been published. We hope that this review will serve to highlight the importance that the global neuroimaging community works toward adopting standards that facilitate searching for and accessing relevant neuroimaging databases. The Brain Research International Data Governance and Exchange (BRIDGE; https://braindatagovernance.org) is a project that started up with the IBI support and is currently funded by the Wellcome Trust. BRIDGE's mission is to work with international partners to develop protocols, and data governance frameworks that will allow international data standardization and sharing and will help streamline technology and regulatory components that currently hinder data transfer, standardization and sharing.

## Conclusions

Here we comprehensively surveyed available MRI databases of psychiatric and neurological disorders worldwide. We hope that this survey is helpful for readers who are involved in the development of disease markers, in basic science to understand disease mechanisms, in making policies for research programs and initiatives, and in funder and government policy. We also expect this review paper will contribute to develop policies for international data governance for neuroscience a task advocated by IBI and currently carried on by BRIDGE for the neuroscience community. Although the importance of large‐scale multi‐center and multi‐disorder databases has been acknowledged for making brain imaging useful in actual clinical practice, the available datasets are still far from optimal. Our ongoing BMB human MRI project will release multi‐center, multi‐disorder data from thousands of patients and will strongly contribute to the development of novel therapies for psychiatric and neurological disorders.

## Disclosure statement

We have read the journal's policy and the authors of this manuscript have the following competing interests: YT was employed by SHIONOGI & CO., Ltd. The remaining authors declare that the research was conducted in the absence of any commercial or financial relationships that could be construed as a potential conflict of interest. Masaru Mimura is the Editorial Board members of Psychiatry and Clinical Neurosciences and the co‐authors of this article. Shinsuke Koike is a Field Editor of section Neuroimaging in Psychiatry and Clinical Neurosciences and a co‐author of this article. To minimize bias, they were excluded from editorial decision‐making related to the acceptance and publication of this article. Hidehiko Takahashi is the Editor‐in‐Chief of Psychiatry and Clinical Neurosciences and a co‐author of this article. Editorial decision‐making was handled independently by Editor‐in‐Chief Tadafumi Kato to minimize bias.

## Author contributions

Conceptualization: SCT, K Kasai, Y Okamoto, SK, OY, TJ, FP, KD, MN, MK, T Hanakawa, Data curation: SCT, Y Okamoto, SK, GO, HS, EI, AT, KI, T Hanakawa. Formal analysis: SCT, SK, Funding acquisition: SCT, K Kasai, Y Okamoto, SK, MN, JM, AS, MK, T Hanakawa, Investigation: SCT, Y Okamoto, SK, GO, HS, EI, YT, AT, TI, RA, YK, MS, JM, SS, M Aki, NO, SM, NS, M Abe, Y Oi, KS, K Kamagata, MH, YA, JH, SH, NN, MF, TN, KI, T Hanakawa, Methodology: SCT, Y Okamoto, SK, T Hanakawa, Project administration: SCT, Y Okamoto, SK, MN, MK, T Hanakawa, Resources: SCT, Y Okamoto, SK, T Hanakawa, Software: SK, Supervision: SCT, K Kasai, Y Okamoto, SK, MN, MM, HT, NH, AS, HI, MK, T Hanakawa, Validation: SCT, SK, Visualization: SCT, Y Okamoto, SK, MAbe, AS, Writing original draft: SCT, Y Okamoto, SK, T Hayashi, AY, OY, TJ, FP, HS, TI, MS, MAbe, YA, AS, T Hanakawa, Writing review & editing: All authors.

## Supporting information


**Data S1.** Supplementary Information.
