# Supplementary material for: The status of MRI databases across the world focused on psychiatric and neurological disorders
Source: Psychiatry Clin Neurosci. 2024 Aug 20;78(10):563–79. doi: 10.1111/pcn.13717 (PMC11804910; doi:10.1111/pcn.13717)

Figure S1

The population of participants in each dataset. “Other” includes patients with disorders other than the six targeted disorders. NA indicates that information about numbers of patients and healthy controls is not available (mixed). The color key is the same as that used in Figure 1. MD, mood disorders; DD, developmental disorders; SCH, schizophrenia; PD, Parkinson’s disease; AD, Alzheimer’s disease; HC, healthy control participants. Numbering corresponds to the number of the list (Table 1-5).


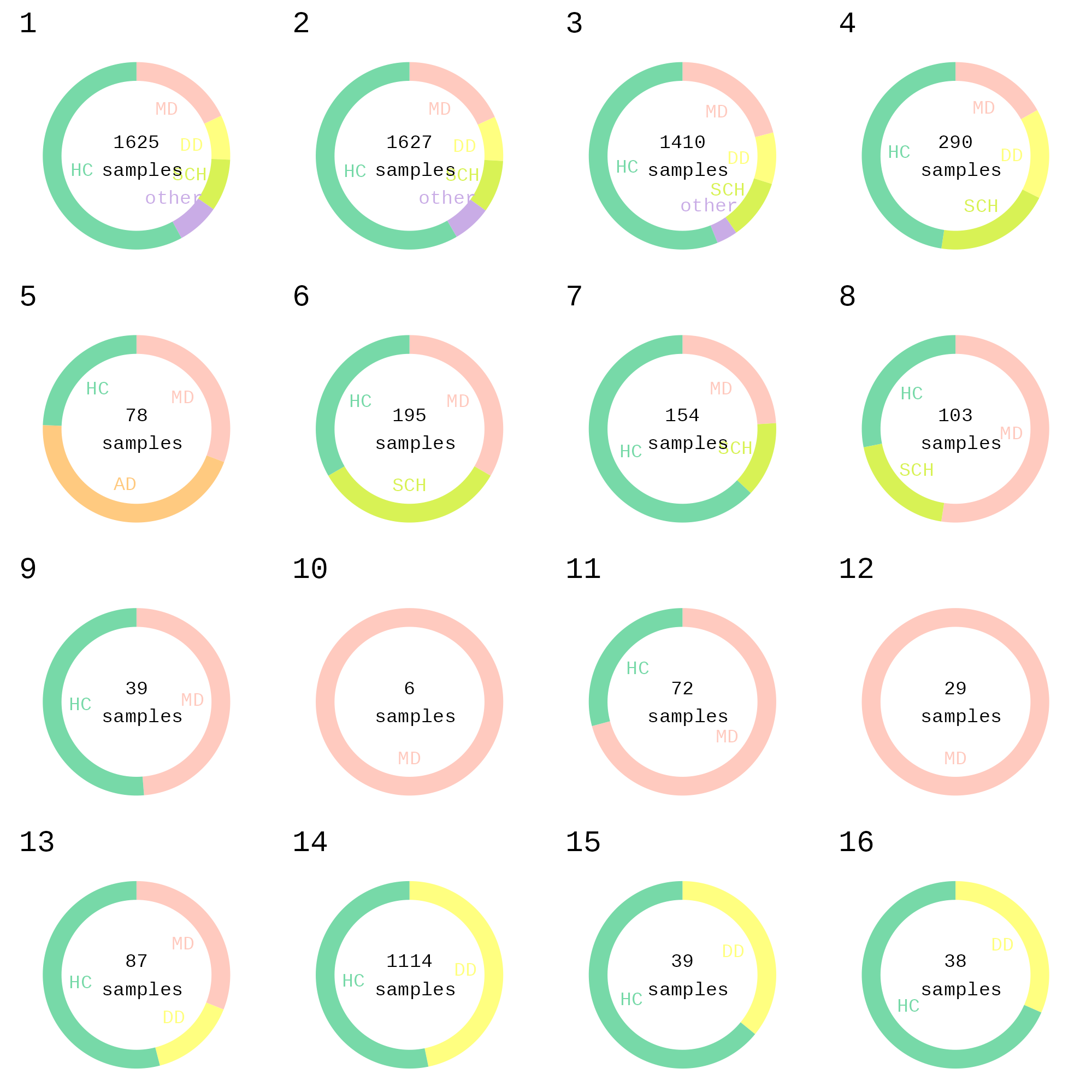


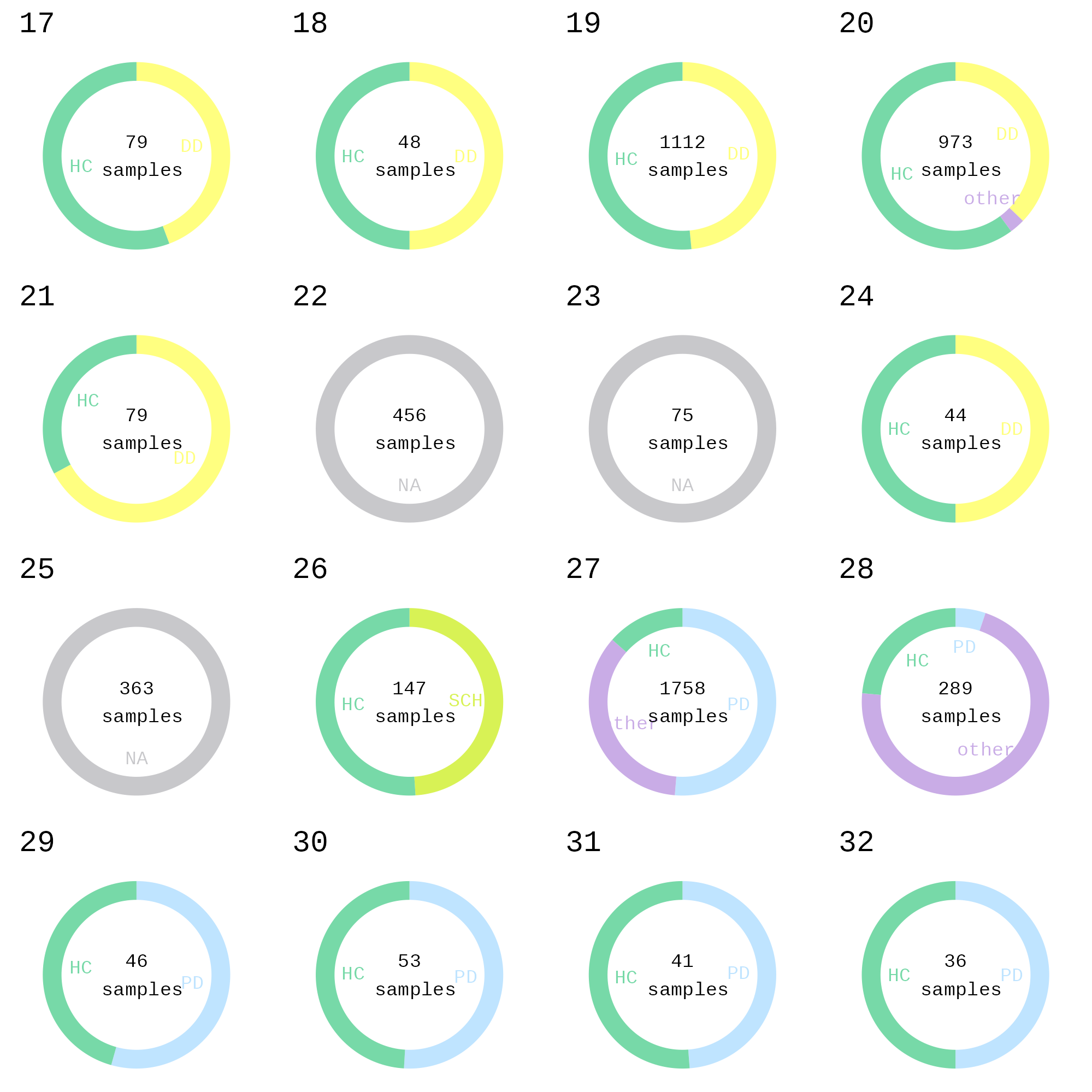


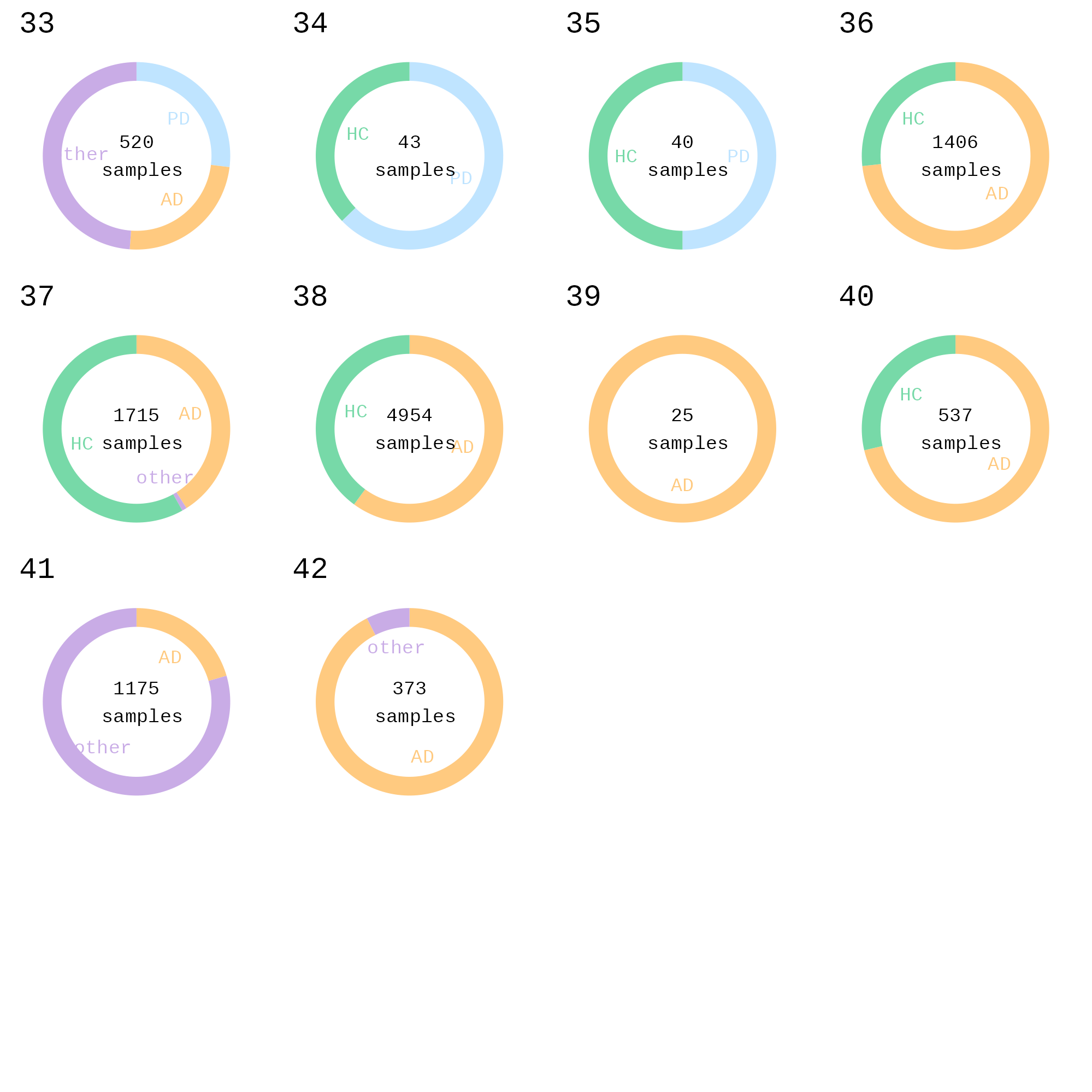

Supplement: Supplementary file 1 — Data S1. Supplementary Information. [file PCN-78-563-s001.docx]
